# Evaluation of the association between long-lasting insecticidal nets mass distribution campaigns and child malaria in Nigeria

**DOI:** 10.1186/1475-2875-12-14

**Published:** 2013-01-09

**Authors:** Hmwe Hmwe Kyu, Katholiki Georgiades, Harry S Shannon, Michael H Boyle

**Affiliations:** 1 Department of Psychiatry and Behavioural Neurosciences, McMaster University and Offord Centre for Child Studies, Hamilton, ON, L8S 4K1, Canada; 2 Department of Clinical Epidemiology & Biostatistics, Faculty of Health Sciences, McMaster University, Hamilton, ON, L8S 4K1, Canada

**Keywords:** Child malaria, Insecticide-treated nets, Campaigns, Evaluation

## Abstract

**Background:**

Nigeria carries the greatest malaria burden among countries in the world. As part of the National Malaria Control Strategic Plan, free long-lasting insecticidal nets (LLINs) were distributed in 14 states of Nigeria through mass campaigns led by different organizations (the World Bank, UNICEF, or the Global Fund) between May 2009 and August 2010. The objective of this study was to evaluate the association between LLIN distribution campaigns and child malaria in Nigeria.

**Methods:**

Data were from the Nigeria Malaria Indicator Survey which was carried out from October to December 2010 on a nationally representative sample of households. Participants were women aged 15–49 years and their children aged less than five years (N = 4082). The main outcome measure was the presence or absence of malaria parasites in blood samples of children (6–59 months).

**Results:**

Compared with children living in communities with no campaigns, those in the campaign areas were less likely to test positive for malaria after adjusting for geographic locations, community- and individual-level characteristics including child-level use of insecticide-treated nets (ITNs). The protective effects were statistically significant for the World Bank Booster Project areas (OR = 0.18, 95% CI = 0.04-0.73) but did not reach statistical significance for other campaign areas. Results also showed that community-level wealth (OR = 0.51, 95% CI = 0.34-0.76), community-level maternal knowledge regarding malaria prevention (OR = 0.70, 95% CI = 0.50-0.97), and child-level use of ITNs (OR = 0.79, 95% CI = 0.63-0.99) were negatively associated with child malaria.

**Conclusions:**

The observed protective effects on child malaria of these campaigns (statistically significant in the World Bank Booster Project areas and non-significant in the other areas) need to be corroborated by future effectiveness studies. Results also show that improving community-level maternal knowledge through appropriate channels might be helpful in preventing child malaria in Nigeria.

## Background

Malaria caused an estimated 655,000 deaths worldwide in 2010 and more than 90% of these deaths occurred in Africa [1]. Nigeria carries the greatest malaria burden among countries in the world with over 300,000 malarial deaths each year - most of them occurring in children under five years of age [2,3]. Malaria is transmitted from person to person by the bite of *Anopheles* mosquitoes. Symptoms appear seven to 30 days after biting by the mosquito that carries the malaria parasite [4]. According to the Nigeria Malaria Indicator Survey (NMIS) [5], almost everyone in the country is at risk for malaria transmission except the minority (3%) located at an altitude 1,200 to 1,400 metres, where the transmission risk is relatively low. The duration of the malaria transmission season decreases from the South to the North [5].

There has been a rapid scale-up of insecticide-treated net (ITN) or long-lasting insecticidal net (LLIN) distribution in African countries in the recent years [1], fuelled by randomized controlled trials (efficacy studies) of ITN on a variety of outcomes. A systematic review of randomized trials reported protective effects of ITN on all-cause mortality in children less than five years of age, anaemia, splenomegaly and prevalence of malaria infection [6]. One randomized trial (conducted after the publication of the systematic review) among children aged six months to five years (N = 100) in a rural community in Nigeria also reported that febrile episodes and parasitaemia were significantly lower in the ITN group compared with the traditional bed net group [7]. While many studies have examined the efficacy of ITNs (under optimal conditions), relatively few studies have looked at the effectiveness of ITN mass distribution campaigns in preventing malaria (under real-world conditions in the general population). A study conducted by Lim and colleagues [8] examined the association between individual-level household ITN ownership or use and malaria parasitaemia among children in the general population in seven countries, but did not look at area-level ITN coverage and reduction in malaria prevalence. In the scale-up of ITN interventions at the national level, using randomized controlled trials to evaluate the effectiveness of the programmes would be unethical since these study designs require withholding of an intervention of proven efficacy [9]. According to Habicht and colleagues [10], one might assume that reduction in disease burdens (e.g. malaria prevalence) are attributable to the interventions (e.g. scale-up of ITNs) after one has attempted to rule out external factors (e.g. improved socioeconomic status, the presence of ancillary projects in the intervention areas, etc.) using non-randomized control groups, which may be either cross-sectional (i.e., comparing intervention and control groups at the end of the programme) or longitudinal (i.e., comparing intervention and control groups both at the beginning and end of the programme). One evaluation study [11] has been conducted in three districts of Togo, comparing changes in malaria morbidity among children before and after an integrated LLIN-measles campaign. This study found protective effects of campaigns in two of the three districts but the lack of programme effects in the remaining district could not be explained.

### Malaria preventive interventions in Nigeria

**Figure 1:**
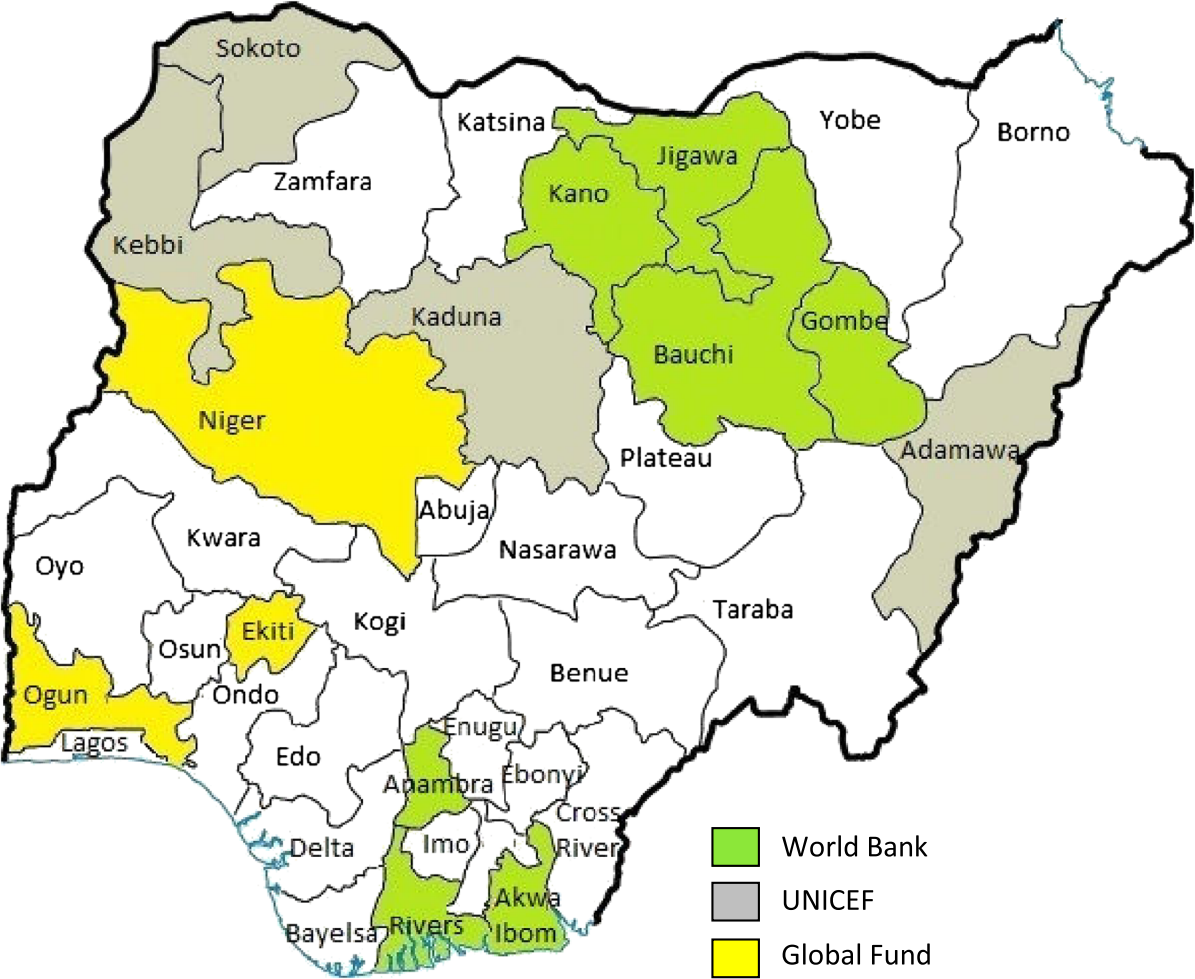
Map of LLIN Distribution Coverage in Nigeria.

The National Malaria Control Strategic Plan (2009–2013) in Nigeria includes universal access to LLINs, increased indoor residual spraying, and environmental management to decrease mosquito breeding places [5,12]. The LLIN distribution strategy in Nigeria included a “scale-up phase” (2009–2010) of free LLIN distributions through mass campaigns (2 LLINs per household); and a “keep-up phase” of replacing “torn or worn out nets” and providing LLINs to new household members and new families [5,12]. Mass distribution of LLINs started in Kano state in May 2009 [3] and more than 24 million LLINs were distributed in 14 of the 37 states in Nigeria by August 2010 [12]. The scale-up phase LLIN distribution strategy was through stand-alone mass distribution campaigns, led by the states in collaboration with partners, using common methodology and tools [13]. The National Malaria Control Programme, partners and stakeholders (i.e., government at all levels, donors, implementers, etc.) coordinate through federal and state LLIN Campaign Coordination Networks [13,14]. Under the networks, there were technical, demand creation, and logistics teams to support the campaign operations in each state [13]. Trained staff went out to the communities, registered each household and provided a net card that could be redeemed for two LLINs at a distribution point which could be primary health centres, schools or homes of community heads [13,15,16]. The campaigns supported by the United Nations Children’s Fund (UNICEF) were integrated with child health interventions [12,15]. In these integrated campaigns, in addition to registering and providing net cards to households, all children under five years of age received polio immunization [15]. RapidSMS (a short message service (SMS) based data collection and communication tool) was used to monitor the distribution of LLINs in all states [14,17]. Lead partners responsible for LLIN distribution include: the World Bank in seven states (Akwa Ibom, Anambra, Bauchi, Gombe, Jigawa, Kano, and Rivers), UNICEF in 4 states (Adamawa, Sokoto, Kaduna, Kebbi) and Global Fund (through the Society for Family Health and Yakubu Gowon Centre) in three states (Niger, Ogun and Ekiti) [5,12,18] (see also Figure 1). Campaigns in these 14 states were completed by August 2010 or earlier [12]. In a separate initiative just before the start of the universal mass distribution campaigns in 14 states, United States Agency for International Development (USAID) and the Canadian Red Cross delivered 676,877 LLINs to children aged less than five years in Cross River State in late 2008 and early 2009 [12]. An evaluation report of the Cross River State campaign showed that 81% of households received an LLIN [12].

The World Bank selected the seven states based on the following criteria [5]: high mortality rates among children aged less than five years (> 260 deaths per 1,000 live births); high prevalence of drug-resistant *Plasmodium falciparum* malaria (> 85%); demonstrated commitment by the states to implement the campaigns; and a lack of substantial aid from other donors to control malaria in the states. No information was available as to the selection of states for campaigns led by UNICEF and Global Fund.

According to the NMIS conducted between October and December 2010, 42% of households reported owning at least one ITN compared with 8% in the 2008 Nigeria Demographic and Health Survey (NDHS) [5,19]. The percentages of households owning at least one ITN in the 14 campaign states (72% in the World Bank Booster areas and 75% in other campaign areas) were more than three times that of households in areas with no campaigns (22%) [5]. Less than 1% of the households were reported to have been sprayed indoors in the past 12 months prior to the survey [5].

### Individual- and community-level influences on malaria infection

Failure to use available nets and lack of knowledge about malaria prevention are important determinants of malaria risk. Despite the ownership of an ITN in households, the NMIS (2010) reported that only 49% of people used them the night before the survey and that use extended to only 59% of children [5]. When asked about various ways to avoid getting malaria, only 17% of women answered “using an ITN or LLIN” while 20% and 8% of women cited “using insecticide spray” and “eliminating stagnant water around living areas”, respectively [5]. Lack of knowledge at the individual and community levels about the effectiveness of preventive interventions may result in refusal to: use an ITN, spray indoors, or participate in neighbourhood clean-up activities to remove mosquito breeding sites, since people may not understand or know the importance of these preventive activities.

In addition to malaria preventive knowledge, other community and individual-level factors may influence risk of malaria infection. According to a study in the Democratic Republic of Congo [20], both community and individual-level wealth were protective against malaria infections among adults. The exact mechanism through which wealth could reduce the prevalence of malaria is not clear but it might be attributable to better housing conditions, better access to health care services, and the ability to purchase anti-malarials that could reduce the duration of infection. In addition, higher levels of education are generally related to wealth and also to knowledge of prevention measures which could in turn be linked to reduced risk of malaria. The same study [20] found that increased community use of bed nets was protective against malaria at the community-level but the effect of individual-level bed net use was not statistically significant. Malaria infection has also been associated with proxies for crowding including household size in rural Benin [21] and the number of persons per sleeping room in a peri-urban area of the Gambia [22]. A study among Nigerian children using the NDHS (2008) found that fever (as a proxy for malaria) was associated positively with the child’s age, being male and living in a rural area [23]. Malaria studies have confirmed the elevated risk of infection associated with rural residency [24,25] and reported a negative association between geographic elevation and the human biting rate of *Anopheles* mosquitoes [26].

### Rationale and study objectives

The previous study in Togo [11] evaluated the impact of integrated LLIN campaigns on malaria morbidity among children less than five years old in three districts of Togo using a before-after design. Adjustment for confounding is crucial in such an approach since there is no external comparison group [10]. The Togo study controlled for age and sex of the child, household socio-economic status, and the education level of the caretaker but there were no attempts to take into account community-level characteristics (e.g. malaria preventive knowledge). In Nigeria, although LLINs have been freely distributed on a large scale among the general population either through standalone or integrated campaigns, no study has attempted to evaluate the impact of these campaigns on malaria infections among children. The availability of the NMIS (2010) data provides an opportunity to conduct a low-cost secondary data analysis to evaluate the impact of LLIN campaigns on child malaria, using an external comparison group (i.e., states that have not been covered by the campaigns) and adjusting for an extensive array of control variables both at the individual- and community-levels. Although this secondary data analysis study can contribute to the understanding of malaria control, dependence on one cross-sectional study to assess ‘outcome’ (parasitological test results for malaria in children) poses a significant challenge, namely, to establish comparability of risk at baseline among the study areas – those selected and not selected for LLIN campaigns. This study attempted to achieve comparability by statistically controlling for population differences in levels of risk using: fever data from the 2008 NDHS [19].

The objectives of this study were: (1) to assess the between-community and between-household variations in malaria among children (in terms of presence of malaria parasites in blood samples) in Nigeria; (2) to examine the association between LLIN distribution campaigns and child malaria after adjusting for geographic characteristics; and (3) to examine if campaign effects are explained by other community and individual-level characteristics.

## Methods

The 2010 Nigeria Malaria Indicator Survey (NMIS) was carried out from October to December, 2010 on a nationally representative sample of households. The objectives of NMIS include (but are not limited to): measuring the ownership and use of bed nets, measuring malaria prevalence among children, and assessing malaria related knowledge, attitude and practices among women. The detailed methodology is available elsewhere [5], and a brief outline is provided here. The sampling frame was a list of census enumeration areas (EAs) from the 2006 Population and Housing Census of the Federal Republic of Nigeria. EAs formed the primary sampling units or clusters. The sample was selected using a stratified, two-stage cluster design consisting of 240 clusters, 83 in the urban areas and 157 in the rural areas. (One cluster was inaccessible resulting in 239 clusters in the final sample). In each cluster, all households were listed and an average of 26 households was selected by equal probability systematic sampling. All women aged 15–49 years in the selected households were eligible to be interviewed. The response rate was 97%. During the interviews, women were asked questions on topics including socio-demographic characteristics and knowledge of malaria symptoms, causes, prevention and treatment. In addition, all children aged 6–59 months in selected households were eligible to be tested for malaria. Of the children eligible for testing, 91% were tested for malaria using blood smears collected for malaria microscopy (the detailed procedure is provided in the description of dependent variable). Verbal and written informed consents were obtained from each participant and from parents or guardians for testing of children. The NMIS was approved by the Nigeria Health Research Ethics Committee of the Federal Ministry of Health.

## Concepts and measures

### Dependent variable

The outcome is the presence or absence of malaria parasites in blood samples of children (6–59 months). Trained laboratory scientists obtained finger (or heel) prick blood samples from eligible children and prepared a thick blood smear and thin blood film for each child to be examined in the Department of Medical Microbiology and Parasitology Laboratory at the University of Lagos. The laboratory had ten experienced malaria microscope specialists and each blood slide was examined by two independent specialists. All discordant results were read and adjudicated by a third specialist.

### Individual-level variables

*Maternal knowledge of malaria prevention:* a knowledge score was based on a count of correct responses coded 1, yes or 0, no to 8 items following the stem question: “What are the ways to avoid getting malaria?” Sample items include: sleep under an ITN/LLIN, use insecticide spray, use mosquito coils, and eliminate stagnant water around living area.

Other individual-level variables included: maternal education in total years of schooling, whether the child slept under an ITN the night before the survey, whether the child was treated with anti-malarial drugs for fever during the past two weeks before the survey, child gender and child age in months (rescaled for the purposes of data analyses so that one unit increase represents one year).

### Household-level variables

*Household wealth:* this study used the wealth index [27] variable already available in the NMIS dataset. It was derived from an index (generated through principal component analysis) of household assets ranging from televisions to bicycles, characteristics of the dwelling, source of drinking water and sanitation facilities. The index was standardized to a mean of 0 and a standard deviation of 1 and higher scores refer to greater wealth.

*Average number of people per sleeping room:* this variable was calculated by dividing the number of household members by the number of rooms used for sleeping in the household.

### Community-level variables

*ITNs coverage*, *proportion of children using ITNs, proportion of febrile children treated with anti-malarial drugs, wealth* and *maternal knowledge of malaria prevention* at the *community level* were calculated by aggregating the corresponding household and individual-level variables (i.e., whether a household has at least one ITN, whether a child slept under an ITN the night before the survey, whether a child with fever was being treated with anti-malarial drugs, wealth index and maternal knowledge) up to the cluster levels.

*Areas for long-lasting insecticidal nets (LLINs) distribution campaigns:* three dummy variables were created based on the main lead partners of the campaigns: “the World Bank Booster Project” “UNICEF”, and “Global Fund”. [Note: In Kaduna State, Global Fund and UNICEF were responsible for distributing 477,649 and 2,253,539 LLINs respectively [18]. Since the former only distributed 17% of total LLINs in Kaduna, this state was classified into the UNICEF group for the purposes of the analyses.

*Time from campaigns to NMIS:* respondents were asked if the household has any mosquito nets. For those who answered ‘yes’ , they were asked where and how many months ago the nets were obtained. Respondents were also asked the type and brand of the net. If they did not know the brand and if the interviewer also could not observe the net, respondents were shown pictures of typical net types/brands. More than 75% of households that have at least one ITN in the campaign areas reported that they received it from net distribution campaigns. The brands of LLINs being reported include Permanet, Olyset, Iconlife, Duranet, and Netprotect. Individual responses on how many months ago the nets were obtained from campaigns were aggregated up to the state level to calculate the *time from campaigns to NMIS*. This variable was then categorized as 3 to 4 months, 5 to 8 months, and 9 to 13 months.

### Geographic variables

*Regions* include: north-central, north-east, north-west (reference), south-east, south, south-west.

*Urban/rural residence* was coded as 0, rural and 1, urban.

*Cluster (EA) altitude in metres* was classified as 200 m or less (reference), 201 to 400m, 401 to 600m, and 601 to < 800m, and more than 1,000m. (There were no clusters at the altitudes between 800 and 1000 metres).

*State-level differences in risk of fever*: prevalence of fever among children 6–59 months in 2008 estimated from NDHS, 2008 [19]. In the absence of baseline information on differences between campaign areas in levels of risk for child malaria, this study obtained state-level estimates of child fever in 2008. In young children, the proportion of fever attributable to malaria is very high during the rainy season in malaria endemic areas [28-30]. The NDHS (2008) was conducted during the rainy season (June to October, 2008), enabling the authors to: (1) estimate campaign-area differences in risk for malaria just before the programmes were launched; and (2) evaluate the ability to control for these differences statistically (i.e., using state-level differences in risk for fever as a control variable to adjust for campaign-area differences in risk for malaria prior to the programmes).

Using the 2008 NDHS to model risk of child fever at the individual level as a function of the campaign areas indicated that there were significant differences between them in fever (as a proxy for malaria risk) compared with non-campaign areas. For example, areas served by the World Bank campaign exhibited elevated risk (OR = 1.56, 95% CI = 1.33-1.82) while the campaigns served by UNICEF (OR = 0.64, 95% CI = 0.52-0.79) and the Global Fund (OR = 0.74, 95% CI = 0.57-0.96) exhibited lower risk compared with non-campaign areas. Controlling for the prevalence of child fever at the state-level explained all these differences: the World Bank Booster areas (OR = 0.97, 95% CI = 0.85-1.11), UNICEF (OR = 1.01, 95% CI = 0.85-1.21) and Global Fund (OR = 1.00, 95% CI = 0.81-1.25).

### Data analysis

Sample weights were used in the descriptive analyses but not in estimating associations as recommended in the Guide to Demographic and Health Survey (DHS) Statistics [31]. According to the Guide [31], use of sample weights is suitable for obtaining representative statistics (e.g. means and percentages) but unsuitable for estimating associations (e.g. regression coefficients) and could also distort variances used to estimate confidence intervals. This study used multi-level modeling and the statistical software MLwiN version 2.26 [32] to conduct a three-level regression analysis of the NMIS data with children nested in households nested in clusters. All estimates were derived by the use of second-order penalized quasi-likelihood and iterative generalized least squares estimation. Residual variation at level 1 is assumed to have a standard logistic distribution with mean zero and variance π^2^/3 =3.29 [33]. At subsequent levels (households, clusters), the variance partition coefficient is given by the estimated residual variation at each level divided by total residual variation [33].

### Sample for analysis

Of the 4,901 children (6–59 months) whose mothers were interviewed, 4,345 children were tested for malaria and had blood test results available. Of the 4345 children, 175 children (4.0%) were either visitors or had missing data on covariates and were excluded. Because the sample size of children (N = 88) in Cross River State (where USAID and the Canadian Red Cross delivered 676,877 LLINs to children aged less than five years in late 2008 and early 2009) was too small to be included as a separate independent variable, these children were excluded from the analysis. The final sample for analysis included 4,082 children.

## Results

Sample characteristics by campaign areas are presented in Additional file 1. The overall sample comprised of 4082 children living in 2549 households in 233 clusters. About 42% of children tested positive for malaria parasites overall: the lowest rate was associated with the World Bank Booster Project (34.4%), and the highest rate, with the Global Fund (62.5%) compared to 41.8% in areas with no campaigns. Among the campaign areas, the Global Fund had the smallest sample of children and the lowest levels of ITN use: 40.2% at the child level, and 34.9% at the community level.

Between-community/cluster and between-household variations (variance partition coefficients) in malaria among children were 2.025 and 0.241 - the corresponding percentages were 36.5% [2.025/(2.025 + 0.241 + 3.29)] and 4.3% [0.241/(2.025 + 0.241 + 3.29)] respectively. The results in two models, each one adding a new group of variables, are summarized in Additional file 2. In Model 1, compared with areas having no campaigns, children living in areas covered by the World Bank Booster Project (OR = 0.18, 95% CI = 0.04-0.79) were less likely to have tested positive for malaria after adjusting for geographic variables, state-level fever rates prior to the start of the campaigns, and time from campaigns to NMIS. In Model 2, after including the community- and individual-level variables, the World Bank effect remained the same and the confidence interval was a bit narrower (OR = 0.18, 95% CI = 0.04-0.73). The campaign areas led by UNICEF and Global Fund exhibited negative associations with child malaria that were not statistically significant. Community-level wealth (OR = 0.51, 95% CI = 0.34-0.76), community-level maternal knowledge regarding malaria prevention (OR = 0.70, 95% CI = 0.50-0.97), and child-level use of ITNs (OR = 0.79, 95% CI = 0.63-0.99) were negatively associated with child malaria.

## Discussion

In this study, compared with children living in areas with no campaigns, those in the World Bank Booster Project areas were less likely to test positive for malaria after adjusting for numerous variables measured at the region, state, community and individual levels. This was also the case for the other campaign areas, although the associations were not statistically significant. Community-level wealth, community-level maternal knowledge regarding malaria prevention, and child-level use of ITNs were negatively associated with child malaria.

The largest validity threats to this study come from baseline differences (non-comparability) between campaign and non-campaign areas in child malaria risk, the relatively small sample coverage in the Global Fund and UNICEF campaign areas and the problems of co-intervention (ancillary programmes operating in campaign areas such as indoor residual spraying campaigns in the World Bank Booster areas [12]) and contamination (programme elements being implemented in non-campaign areas, for example ITNs obtained from a primary health centre or purchased from markets [5]). In addition to accounting for geographic variation in risk associated with regional differences and location (urban–rural residency and altitude), this study controlled for state-level differences in child fever in 2008 before the campaigns began. This study can show that controlling for fever at the state level made the campaign areas comparable in 2008 but cannot be sure that similar conditions of area risk applied in 2009 and 2010: malaria risk varies not only from place-to-place but from one year to the next [34].

Overall, it appears that the campaigns were implemented fairly well. Between 2008 and 2010, household ownership of ITNs increased considerably from 8 to 42% [5,19]. Households in the 14 states with an LLIN campaign were about three times more likely to have at least one ITN than households in states without any LLIN campaign (> 70% in campaign areas versus 22% in areas with no campaigns) [5]. The NMIS (2010) also reported an increase in the proportion of children with fever being treated with anti-malarial drugs from 33% in 2008 to 49% in 2010 [5]. Given that the proportions of children receiving anti-malarial treatment in the World Bank Booster areas (52.4%) and non-campaign areas (50%) (Additional file 1) were quite similar, the observed protective effect against malaria in the World Bank Booster areas was unlikely due to treatment differences. The reduced risk for malaria in these areas may also be attributable to indoor residual spraying but less than 1% of households reported being sprayed in the 12 months prior to the survey (1.7% in the World Bank Booster areas versus 0.4% in non-campaign areas) [5]. When a separate analysis was conducted by restricting the analysis to children who lived in households that were not sprayed, the results were not affected. The observed reduced risk of malaria among children in the World Bank Booster areas could therefore be attributable to the increased use of LLINs in the project areas after the campaigns. Nevertheless, the campaign effects in other campaign areas were less clear cut and a larger sample would be needed to demonstrate statistically significant campaign effects in these areas. Despite a dramatic increase in households’ net ownerships in campaign areas, the take-up of bed nets is relatively lower in the Global Fund areas (34.9%) than the campaign areas led by UNICEF (60.7%) and the World Bank (51.6%).

Failure to use ITNs as planned is a challenge to malaria prevention programmes. According to a survey of 160 randomly selected households in two local government areas in Cross River State conducted by International Federation of the Red Cross in July and August, 2010, 32% of respondents reported that they no longer had the LLINs distributed by the campaigns. Reasons provided include: torn nets; nets had been given out; or nets were used for other purposes (e.g. door screens or bed sheets) [35]. Focus group discussions following the household survey found that a majority of respondents thought the LLINs were no longer effective after a year or the net was not usable after washing twice [35]. Despite the net ownership, misperceptions and lack of awareness about proper maintenance might attenuate the campaign effect. It would be helpful to conduct similar qualitative studies in other campaign areas to better understand the findings of the present study.

In this study, children living in communities with higher levels of maternal knowledge were less likely to have malaria but the association for individual-level maternal knowledge did not reach statistical significance. This finding is plausible if community-level knowledge motivates participation in community-based malaria prevention activities including elimination of mosquito breeding sites in the neighborhood. This study also found that children living in wealthier communities were less likely to have malaria. This finding is consistent with the result of a previous study conducted among adults in the Democratic Republic of Congo [20]. The protective effects of wealth might be attributable to better housing conditions, better access to health care services, higher levels of education and preventive knowledge and the ability to purchase antimalarials that could reduce the duration of malaria infection. Individual-level child ITN use was negatively associated with malaria which is in line with the increased use of ITNs and reduced risk of malaria in the World Bank Booster areas.

The NMIS (2010) is the first of its kind to be conducted in all 36 states and the Federal Capital Territory of Nigeria, and included testing for malaria among children (6–59 months). Because this is a one-time cross-sectional study, the study was unable to assess change and had to rely on statistically controlling for differences between campaign areas in malaria risk that could confound the results. Furthermore, information on programme implementation was limited to maternal report on ITN use. These important methodological limitations are set against several study strengths that includes an attempt to control for baseline differences, using an extensive array of control variables; the incorporation of different campaigns into the analysis; the availability of an external control group; and the low cost of secondary data analysis. The study does provide some insights into effectiveness questions bearing on the prevention of malaria infection among children in the general population.

## Conclusions

The findings of this study show that children living in the World Bank Booster Project areas were less likely to have tested positive for malaria. There was also an observed but statistically non-significant reduction in risk associated with the UNICEF and Global Fund campaigns. These beneficial effects need to be corroborated by future studies, using stronger designs and larger samples. Finally, it is very clear that community level wealth and knowledge exhibit strong negative associations with child malaria. Results also suggest that improving community-level maternal knowledge through appropriate channels might be helpful in preventing child malaria in Nigeria.

## Abbreviations

LLIN: Long-lasting insecticidal net; ITN: Insecticide-treated net; NMIS: Nigeria Malaria Indicator Survey; NDHS: Nigeria Demographic and Health Survey; DHS: Demographic and Health Survey.

## Competing interests

The authors declare that they have no competing interests.

## Authors’ contributions

All authors conceived the study. HK and MB analysed the data and all authors interpreted the results and drafted the manuscript. All authors read and approved the final manuscript.

## Supplementary Material

Additional file 1 Sample characteristics by campaign areas. Click here for file

Additional file 2 Multilevel logistic regressions of child malaria on study variables and covariates. Click here for file
