# Supplementary material for: Evaluation of the association between long-lasting insecticidal nets mass distribution campaigns and child malaria in Nigeria
Source: Malar J. 2013 Jan 9;12:14. doi: 10.1186/1475-2875-12-14 (PMC3545742; doi:10.1186/1475-2875-12-14)
Supplement: Additional file 1 — Sample characteristics by campaign areas. [file 1475-2875-12-14-S1.doc]

**Additional file 1: Sample characteristics by campaign areas**

|  | **World Bank Booster Project** | **UNICEF** | **Global Fund** | **Areas with no campaigns** | **Overall sample** |
| --- | --- | --- | --- | --- | --- |
| Level 3, clusters (n) | 54 | 23 | 15 | 141 | 233 |
| Average number of households per cluster (SD) | 12.1(3.9) | 13.2(3.4) | 10.7(4.7) | 10.2(4.6) | 10.9 (4.4) |
| Average number of children per cluster (SD) | 20.2(7.7) | 21.6(7.1) | 17.6(9.6) | 15.8(8.0) | 17.5 (8.2) |
| Regions (%)  North Central | 0 | 0 | 46.7 | 23.4 | 17.2 |
| North East | 25.9 | 30.4 | 0 | 13.5 | 17.2 |
| North West | 25.9 | 69.6 | 0 | 7.1 | 17.2 |
| South East | 16.7 | 0 | 0 | 22.0 | 17.2 |
| South | 31.5 | 0 | 0 | 12.8 | 15.0 |
| South West | 0 | 0 | 53.3 | 21.3 | 16.3 |
| Urban (%) | 29.6 | 26.1 | 26.7 | 37.6 | 33.9 |
| Cluster altitude (%)  ≤ 200 metre | 44.4 | 4.3 | 46.7 | 47.5 | 42.5 |
| 201-400 metre | 16.7 | 47.8 | 20.0 | 31.9 | 29.2 |
| 401-600 metre | 37.0 | 17.4 | 33.3 | 13.5 | 20.6 |
| 601 to 800 metre | 1.9 | 30.4 | 0 | 2.8 | 5.2 |
| > 1000 metre | 0 | 0 | 0 | 4.3 | 2.6 |
| State-level fever rate (NDHS 2008) *Mean* (SD) | 23.1(10.5) | 11.0(1.8) | 12.9(4.5) | 16.5(8.9) | 16.9(9.0) |
| LLINs distribution campaigns (%)  World Bank Booster Project | 100 | 0 | 0 | 0 | 23.2 |
| UNICEF | 0 | 100 | 0 | 0 | 9.9 |
| Global Fund | 0 | 0 | 100 | 0 | 6.4 |
| No campaigns | 0 | 0 | 0 | 100 | 60.5 |
| Time from campaigns to NMIS (%)  3-4 months | 0 | 50.0 | 0 | NA | NA |
| 5-8 months | 71.4 | 50.0 | 33.3 | NA | NA |
| 9-13 months | 28.6 | 0 | 66.7 | NA | NA |

**Additional file 1 (continued)**

|  | **World Bank Booster Project** | **UNICEF** | **Global Fund** | **Areas with no campaigns** | **Overall sample** |
| --- | --- | --- | --- | --- | --- |
| Community level ITN coverage (% households with at least one ITN) *Mean* (SD) | 72.4(18.5) | 77.1(19.2) | 69.6(22.8) | 22.8(22.1) | 42.7(32.4) |
| Proportion of community-level child ITN use *Mean* (SD) | 51.6(21.5) | 60.7(19.3) | 34.9(25.7) | 13.7(20.4) | 28.5 (28.2) |
| Proportion of children with fever treated with anti-malarial drugs *Mean* (SD) | 52.4(34.2) | 54.7(34.9) | 59.9(36.7) | 50.0(35.3) | 51.6(35.0) |
| Community-level wealth *Mean* (SD) | -0.1(0.9) | -0.5(0.6) | 0.3(0.9) | 0.2(0.9) | 0.1 (0.9) |
| Community-level maternal knowledge *Mean* (SD) | 1.7(0.7) | 2.1(0.9) | 1.2(0.7) | 1.5(0.7) | 1.6 (0.8) |
| Level 2, households (n) | 653 | 304 | 161 | 1431 | 2549 |
| Average number of children per household (SD) | 1.7(0.8) | 1.6(0.8) | 1.7(0.9) | 1.6(0.8) | 1.6 (0.8) |
| Household wealth *Mean* (SD) | -0.3(0.9) | -0.6(0.5) | -0.1(1.0) | -0.1(1.00) | -0.2(1.0) |
| Average number of people per sleeping room (SD) | 3.1(1.4) | 3.3(1.6) | 2.7(1.3) | 3.0(1.4) | 3.0 (1.5) |
| Level 1, children (n) | 1092 | 497 | 264 | 2229 | 4082 |
| Child slept under an ITN (%) | 53.0 | 58.7 | 40.2 | 14.3 | 31.0 |
| Maternal education (yrs) *Mean* (SD) | 3.6(5.2) | 1.7(3.8) | 3.7(5.3) | 5.0(5.1) | 4.2 (5.1) |
| Maternal knowledge *Mean* (SD) | 1.8(1.3) | 1.8(1.2) | 1.1(0.8) | 1.4(1.2) | 1.5(1.2) |
| Child age (yrs) | 2.6(1.3) | 2.6(1.3) | 2.6(1.3) | 2.7(1.3) | 2.6 (1.3) |
| Male child (%) | 53.1 | 50.8 | 48.2 | 50.5 | 51.0 |
| Malaria (%) | 34.4 | 40.9 | 62.5 | 41.8 | 41.6 |

NA**:** Not Applicable
