# Supplementary material for: Evaluation of the association between long-lasting insecticidal nets mass distribution campaigns and child malaria in Nigeria
Source: Malar J. 2013 Jan 9;12:14. doi: 10.1186/1475-2875-12-14 (PMC3545742; doi:10.1186/1475-2875-12-14)
Supplement: Additional file 2 — Multilevel logistic regressions of child malaria on study variables and covariates. [file 1475-2875-12-14-S2.doc]

**Additional file 2:** Multilevel logistic regressions of child malaria on study variables and covariates

|  | Model 1  OR (95%CI) | Model 2  OR (95%CI) |
| --- | --- | --- |
| **Fixed effects**  Level 3 (Cluster)  Regions  North Central | 1.19(0.50-2.82) | 1.61(0.71-3.65) |
| North East | **0.45(0.22-0.94)** | **0.47(0.24-0.93)** |
| North West | Ref. | Ref. |
| South East | 0.44(0.18-1.06) | 1.26(0.51-3.11) |
| South | 0.64(0.26-1.54) | 2.16(0.90-5.17) |
| South West | 0.80(0.31-2.05) | 1.91(0.72-5.09) |
| Urban | **0.26(0.17-0.39)** | 0.68(0.43-1.07) |
| Cluster altitude  ≤ 200 metre | Ref. | Ref. |
| 201-400 metre | 1.48(0.84-2.58) | 1.22(0.73-2.06) |
| 401-600 metre | 1.55(0.81-2.96) | 1.70(0.93-3.12) |
| 601 to 800 metre | 1.40(0.48-4.04) | 1.34(0.51-3.54) |
| > 1000 metre | 0.36(0.08-1.53) | **0.22(0.06-0.87)** |
| State-level fever rates (NDHS 2008) | 1.19(0.86-1.64) | 0.98(0.72-1.33) |
| State-level time from LLIN campaigns to NMIS  3-4 months | Ref. | Ref. |
| 5-8 months | 3.28(0.87-12.38) | 2.84(0.80-10.07) |
| 9-13 months | 3.14(0.60-16.43) | 3.92(0.86-17.83) |
| LLIN distribution campaigns  World Bank Booster Project | **0.18(0.04-0.79)** | **0.18(0.04-0.73)** |
| UNICEF | 0.43(0.17-1.09) | 0.48(0.19-1.25) |
| Global Fund | 0.65(0.11-3.86) | 0.55(0.10-3.07) |
| No campaigns | Ref. | Ref. |
| Community-level ITN coverage |  | 0.96(0.84-1.10) |
| % Children using ITN |  | 1.10(0.95-1.27) |
| % Children with fever treated with anti-malarials |  | 0.99(0.94-1.06) |
| Community-level wealth |  | **0.51(0.34-0.76)** |
| Community-level maternal knowledge |  | **0.70(0.50-0.97)** |
| Level 2 (Household)  Household wealth |  | 0.81(0.66-1.00) |
| Number of people per sleeping room |  | 0.98(0.92-1.04) |
| Level 1 (Child)  Maternal knowledge |  | 0.97(0.88-1.06) |
| Maternal education |  | 0.98(0.95-1.01) |
| Child slept under an ITN |  | **0.79(0.63-0.99)** |

**Additional file 2 (continued**)

|  | Model 1  OR (95%CI) | Model 2  OR (95%CI) |
| --- | --- | --- |
| Level 1 (Child) (continued)  Treated with anti-malarial drugs  Child with fever not being treated |  | Ref. |
| Child with fever being treated |  | 0.98(0.74-1.29) |
| No fever |  | **0.66(0.53-0.83)** |
| Child age |  | **1.30(1.23-1.39)** |
| Male child |  | 1.15(0.97-1.35) |
| **Random effects variances (SE)**  Level 3, cluster | **1.30(0.17)** | **0.99(0.14)** |
| Level 2, household | **0.52(0.11)** | **0.55(0.12)** |
